# Supplementary material for: A CCL2+DPP4+ subset of mesenchymal stem cells expedites aberrant formation of creeping fat in humans
Source: Nat Commun. 2023 Sep 20;14:5830. doi: 10.1038/s41467-023-41418-z (PMC10511504; doi:10.1038/s41467-023-41418-z)
Supplement: Supplementary file 1 — Supplementary Information [file 41467_2023_41418_MOESM1_ESM.pdf]

Supplementary Materials for

A CCL2$^+$DPP4$^+$ subset of mesenchymal stem cells expedites aberrant formation of creeping fat in humans

Fengfei Wu[1], Fangting Wu[1], Qian Zhou[1], Xi Liu[1], Jieying Fei[1], Da Zhang[1], Weidong Wang[1], Yi Tao[1], Yubing Lin[1], Qiaoqiao Lin[1], Tianyan Xu[1], Xinghua Pan[2], Kai Sun[3], Fang Xie[1*] & Lan Bai[1*].

Correspondence to: Fang Xie (stellaff@126.com), Lan Bai (bailan_99@yeah.net)

Supplementary figures

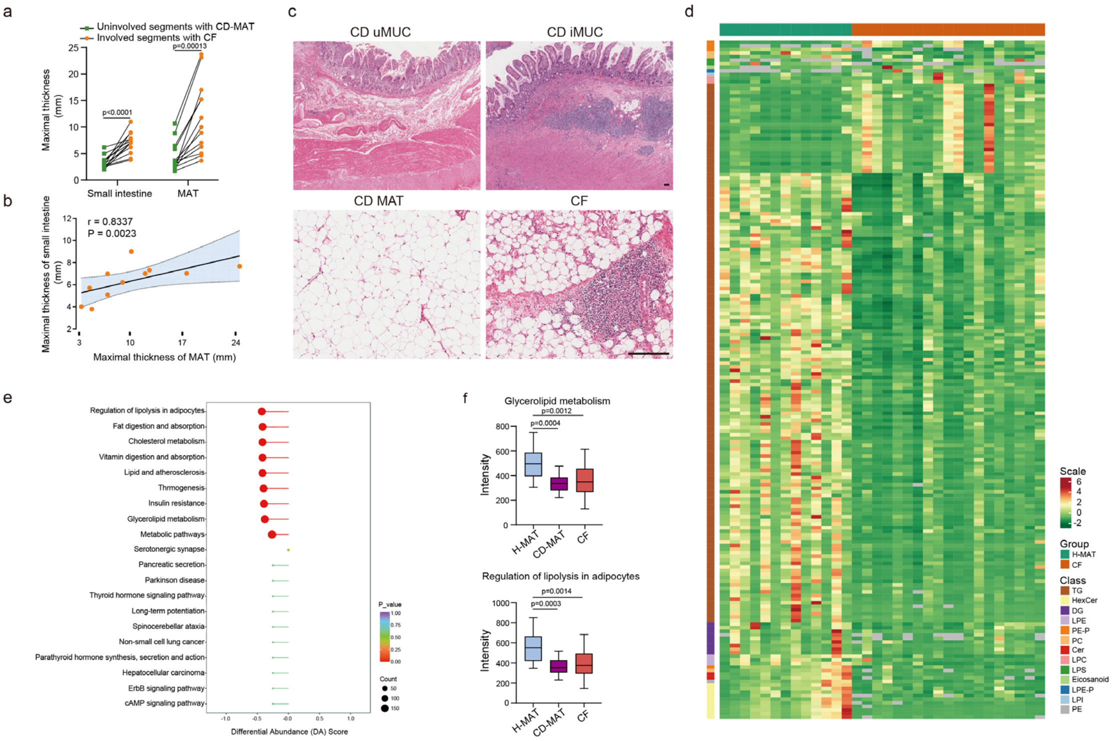

Supplementary Figure 1. Differential lipidomics profiles of H-MAT and CF.

a The maximal thickness of small intestine wall and attached MAT of CD patients (n=13) and p value were analysed using two-sided paired t test. **b** The maximal thickness of MAT or CF plotted against the maximal thickness of corresponding intestinal wall from 11 individuals. Linear regression and Spearman's correlation analysis were conducted. Error bands represent a confidence level of 0.95. $p < 0.05$ was considered significant. **c** H&E stained paired uninvolved and adjacent involved ileal segments (CD uMUC and iMUC, respectively) with attached uninvolved mesenteric adipose (CD MAT) and adjacent CF (representative of 5 BRs per group). Scale bars, 200 μM. **d** Heatmaps of the differences between H-MAT and CF. Data in the heatmap are log2 FC values for each individual lipid. The lipids are sorted within each subclass level by the increasing total number of carbons in the hydrocarbon chains and then the total of double bonds. **e** DA score of the differences in KEGG pathway activities between H-MAT and CF. The size of the circle represents the number of differential metabolites in the pathway and the length of the line segment represents the absolute value of the DA score which indicates the degree of decrease or increase and p value were analysed using hypergeometric test. **f** The metabolite intensity enriched in glycerolipid metabolism and regulation of lipolysis in the adipocytes pathway of H-MAT (n=15), CD-MAT (n=18) and CF (n=20). For boxplot the center line represents the median, box hinges represent first and third quartiles and whiskers represent ± 1.5x interquartile range and p value were analysed using two-sided one-way ANOVA. TG = triglycerides; HexCer =glucosyl- or galactosylceramides; DG = diglycerols; LPE = lysophosphatidylethanolamines; PE-P =Phosphatidylethanolamine; PC = phosphatidylcholines; Cer = ceramides; LPC = lysophosphatidylcholines; LPS= lysophosphatidylserines; LPE-P =lysophosphatidylethanolamines; LPI = lysophosphatidylinositols; PE = phosphatidylethanolamines. Source data are provided as a Source Data file.

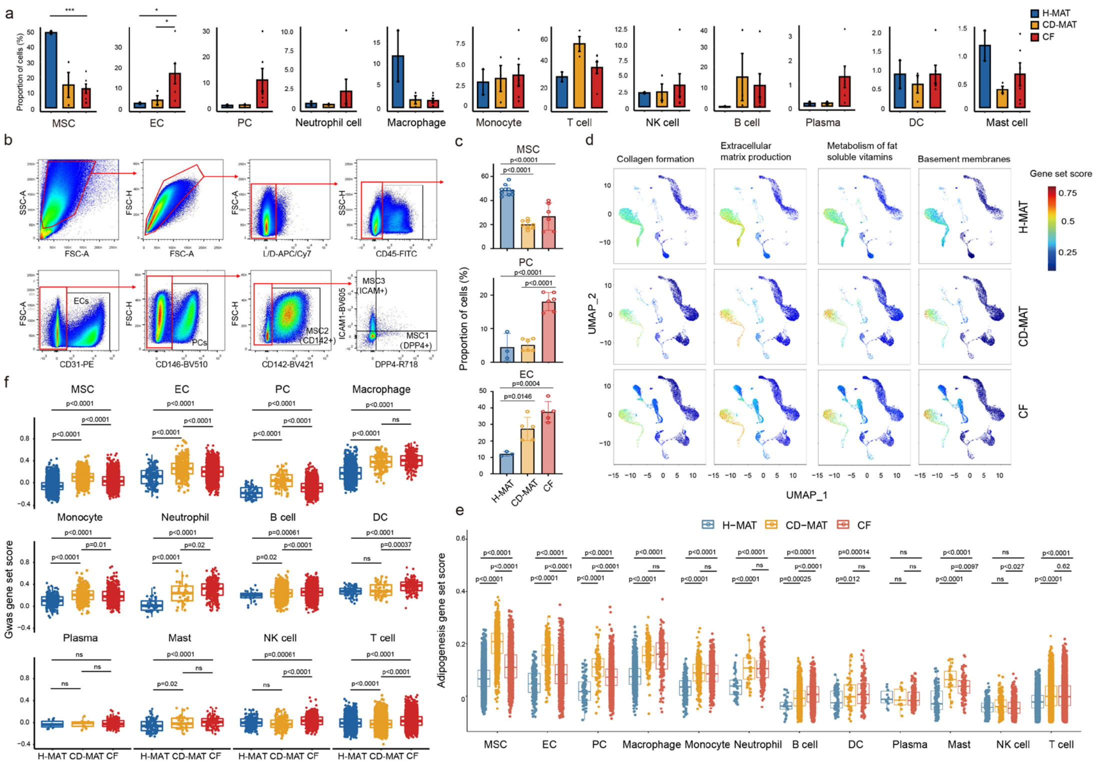

Supplementary Figure 2. Dynamic restructuring of cells in CD.

a Changes in selected cells composition in H-MAT (n=2), CD-MAT (n=3) and CF (n=6). **b** Representative gating strategy for scRNAseq-defined human MAT cells: $CD45^{-}$ cells were stained with anti-CD31, anti-CD146, anti-DPP4, anti-ICAM1, and anti-CD142 antibodies. Endothelial cells ($CD31^{+}$) were selected first, then pericytes ($CD31^{-}$, $CD146^{+}$) were selected, and MSC2 cells ($CD146^{-}$, $CD142^{+}$) were selected subsequently, followed by MSC1 cells ($CD142^{-}$, $ICAM1^{-}$, $DPP4^{+}$) and MSC3 cells ($CD142^{-}$, $DPP4^{-}$, $ICAM1^{+}$). FSC-A: forwards scatter area, FSC-H: forwards scatter height, SSC-H: side scatter height. **c** Flow cytometry showing the percentage of MSCs, PCs and ECs isolated from H-MAT, CD-MAT and CF of control subjects (n=10) and CD patients (n=8). The data representing mean ±s.d. and p value were generated using two-sided one-way ANOVA. **d** Individual cell AUC score overlay for selected canonical pathway activities. Color saturation indicates the gene set score. **e** Boxplots comparing the scores of the adipogenesis gene set in selected cells in H-MAT (n=2), CD-MAT (n=3) and CF (n=6). **f** Boxplots comparing the scores of the GWAS gene set in selected cells in H-MAT (n=2), CD-MAT (n=3) and CF (n=6). For boxplot the center line represents the median, box hinges represent first and third quartiles and whiskers represent ± 1.5x interquartile range. **(e,f)** Data were analysed using two-sided Wilcoxon rank sum test. Source data are provided as a Source Data file.

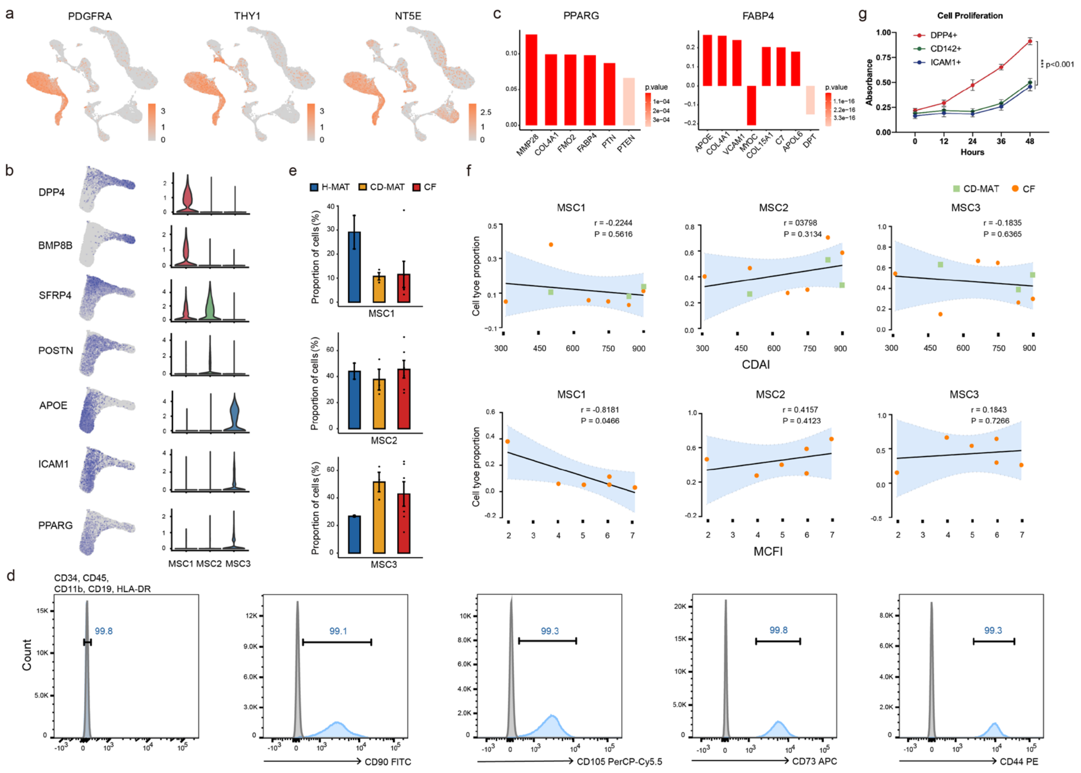

Supplementary Figure 3. Identification of MSC subpopulations.

a Expression levels of representative MSC marker genes in each subpopulation are plotted onto the UMAP. Color key from gray to red indicates relative expression levels from low to high. **b** Individual gene UMAP and violin plots showing the expression levels and distribution representative of marker genes of three MSCs. The y-axis represents log-scaled normalized UMI counts. **c** Correlation (Spearman's rho) between the expression of MSC3 marker genes and PPARG (left) or FABP4 (right). **d** Phenotype of human MSCs showing >99% positivity for CD90, CD105, CD73 and CD44, and no expression of CD34, CD11b, CD19, CD45 or HLA- DR. Flow cytometry results representative of 5 BRs. **e** Changes in selected MSC subpopulations composition in H-MAT (n=2), CD-MAT (n=3) and CF (n=6). **f** Estimated proportions of MSC subpopulations in scRNA-seq data of CD-MAT or CF from 6 individuals plotted against CDAI or MCFI. Linear regression and Spearman's correlation analysis were conducted. Error bands represent a confidence level of 0.95. $p < 0.05$ was considered significant. **g** Quantification of cellular growth (representative of 6 BRs). The data are presented as means ± s.d. and p value were analysed using two-sided one-way ANOVA. Source data are provided as a Source Data file.

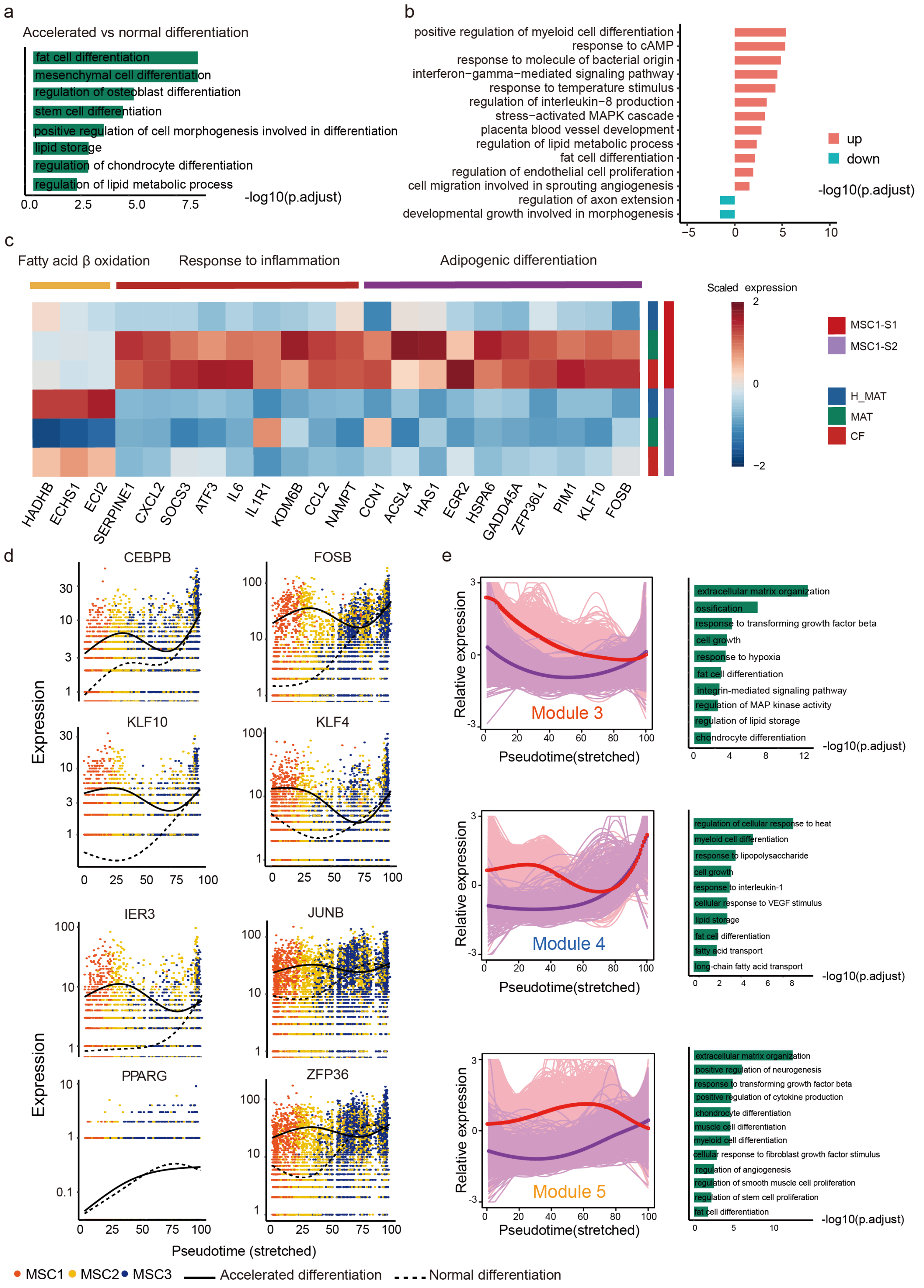

Supplementary Figure 4. Phenotypic characterization of MSC1-S1.

a GO analysis of the differentially expressed genes between accelerated and normal differentiation trajectories based on functions. **b** GO analysis of the upregulated and downregulated differentially expressed genes in MSC1-S1 based on functions. **c** Heatmap depicting canonical adipogenesis-, inflammation-, and metabolism-related genes. **d** Gene expression kinetics along pseudotime progression of representative differentially expressed genes between accelerated and normal

differentiation in (Figure. 4A). Solid line: accelerated differentiation, dashed line: normal differentiation. **e** Spline curve fitted to averaged expression of all genes in module along the MSC1-S2 to MSC3 (red curve) and MSC1-S1 to MSC3 (purple curve) pseudotemporal trajectories (left), with selected enrichment of Gene Ontology terms (right). (**a,b,e)** Data were analysed using two-sided Wilcoxon rank sum test.

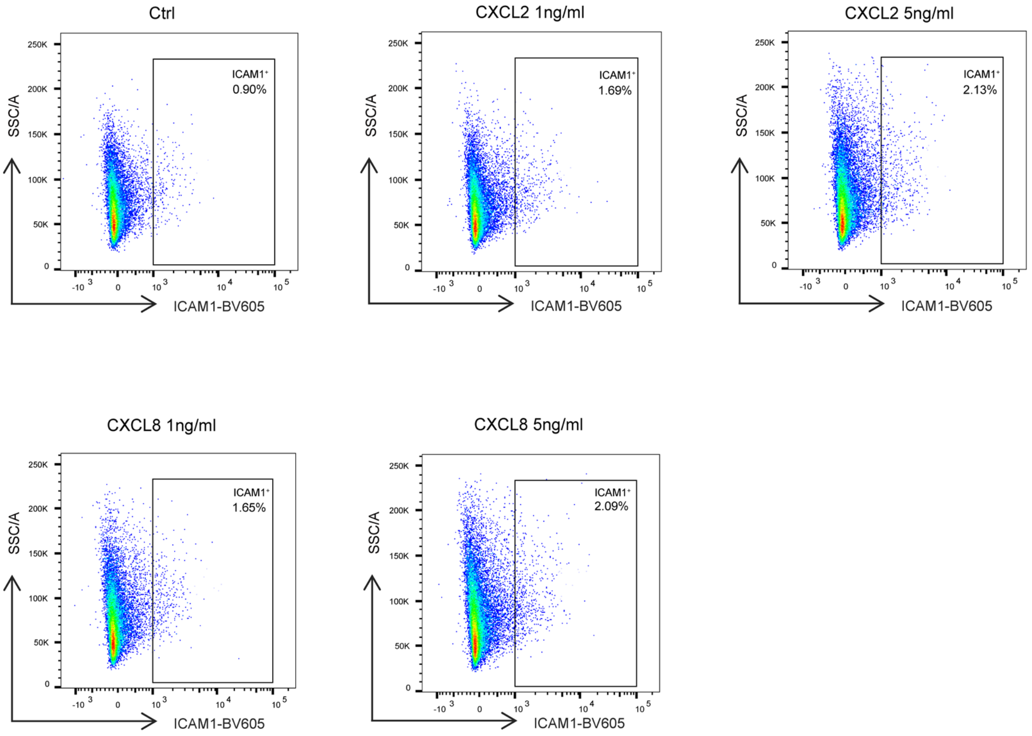

Supplementary Figure 5. Phenotypic characterization of MSC1-S1.

Flow cytometry showing ICAM1 (also known as $CD54^{+}$) expression in MSC1 cells after stimulation with CXCL2 or CXCL8 (1 ng/mL or 5 ng/mL) for 12 hours.

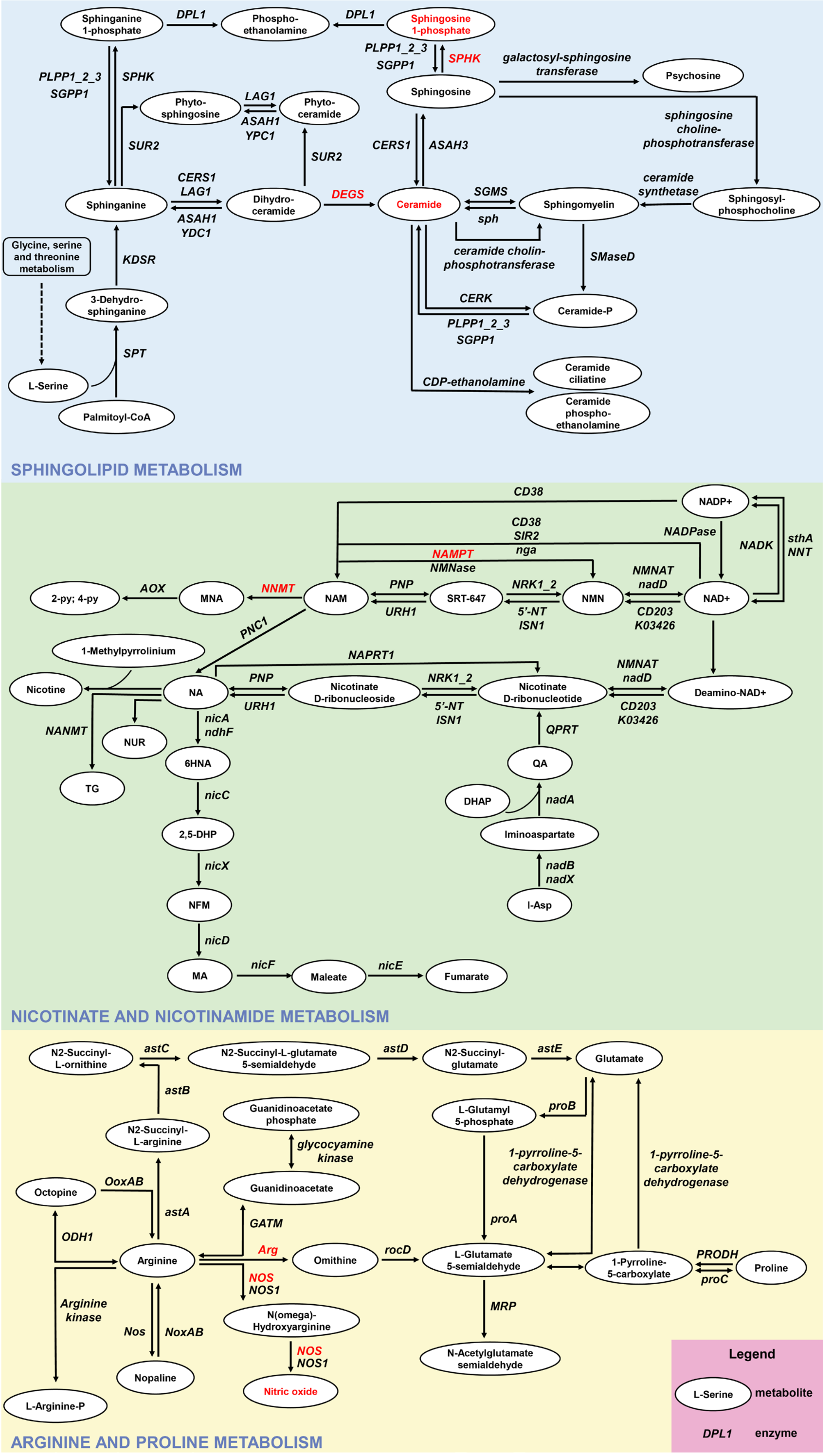

Sphinganine 1-phosphate
DPL1
Phospho-ethanolamine
DPL1
Sphingosine 1-phosphate
PLPP1_2_3
SGPP1
SPHK
galactosyl-sphingosine transferase
Psychosine
PLPP1_2_3
SGPP1
SPHK
Phyto-sphingosine
LAG1
Phyto-ceramide
ASAH1
YPC1
Sphingosine
SUR2
SUR2
sphingosine choline-phosphotransferase
CERS1
ASAH3
CERS1
LAG1
ceramide synthetase
Sphinganine
Dihydro-ceramide
DEGS
Ceramide
SGMS
Sphingomyelin
Sphingosyl-phosphocholine
ASAH1
YDC1
sph
Glycine, serine and threonine metabolism
KDSR
ceramide cholin-phosphotransferase
SMaseD
3-Dehydro-sphinganine
CERK
Ceramide-P
PLPP1_2_3
SGPP1
SPT
L-Serine
Ceramide ciliatine
CDP-ethanolamine
Palmitoyl-CoA
Ceramide phospho-ethanolamine
SPHINGOLIPID METABOLISM
CD38
NADP+
CD38
SIR2
nga
NADPase
NADK
sthA
NNT
NAMPT
NMNase
NMNAT
nadD
2-py; 4-py
AOX
MNA
NNMT
NAM
PNP
SRT-647
NRK1_2
NMN
NAD+
URH1
5'-NT
ISN1
CD203
K03426
PNC1
1-Methylpyrrolinium
NAPRT1
NMNAT
nadD
Nicotine
NA
PNP
Nicotinate D-ribonucleoside
NRK1_2
Nicotinate D-ribonucleotide
Deamino-NAD+
URH1
5'-NT
ISN1
CD203
K03426
NANMT
NUR
nicA
ndhF
QPRT
6HNA
QA
TG
nicC
DHAP
nadA
2,5-DHP
Iminoaspartate
nicX
nadB
nadX
NFM
l-Asp
nicD
MA
nicF
Maleate
nicE
Fumarate
NICOTINATE AND NICOTINAMIDE METABOLISM
N2-Succinyl-L-ornithine
astC
N2-Succinyl-L-glutamate 5-semialdehyde
astD
N2-Succinyl-glutamate
astE
Glutamate
astB
Guanidinoacetate phosphate
L-Glutamyl 5-phosphate
proB
N2-Succinyl-L-arginine
glycocyamine kinase
1-pyrroline-5-carboxylate dehydrogenase
1-pyrroline-5-carboxylate dehydrogenase
Octopine
OoxAB
Guanidinoacetate
astA
GATM
proA
ODH1
Arginine
Arg
Ornithine
rocD
L-Glutamate 5-semialdehyde
1-Pyrroline-5-carboxylate
PRODH
Proline
proC
NOS
NOS1
Arginine kinase
N(omega)-Hydroxyarginine
MRP
Nos
NoxAB
NOS
NOS1
N-Acetylglutamate semialdehyde
Legend
Nopaline
Nitric oxide
L-Serine
metabolite
L-Arginine-P
DPL1
enzyme
ARGININE AND PROLINE METABOLISM

Supplementary Figure 6. Metabolic pathways upregulated in MSC1 and/or cMo-1 cells and were positively associated with CD activities. The pathways included arginine and proline metabolism (at the bottom), nicotinate and nicotinamide metabolism (at the middle) and sphingolipid metabolism (at the top). Metabolites and enzymes marked in red are positively associated with CD activities and facilitate adipogenesis. Sphingosine 1-phosphate (S1P), sphingosine kinase (SPHK), ceramide, nicotinamide phosphoribosyltransferase (NAMPT), nicotinamide N-methyltransferase (NNMT), arginase (Arg) and nitric oxide are potential targets in the treatment of CD. Sphingolipid 4-desaturase/C4-monooxygenase (DEGS) is positively associated with adipocyte differentiation. Expression of nitric oxide synthases (NOS) is closely related to infiltrating cMo-1 cells.

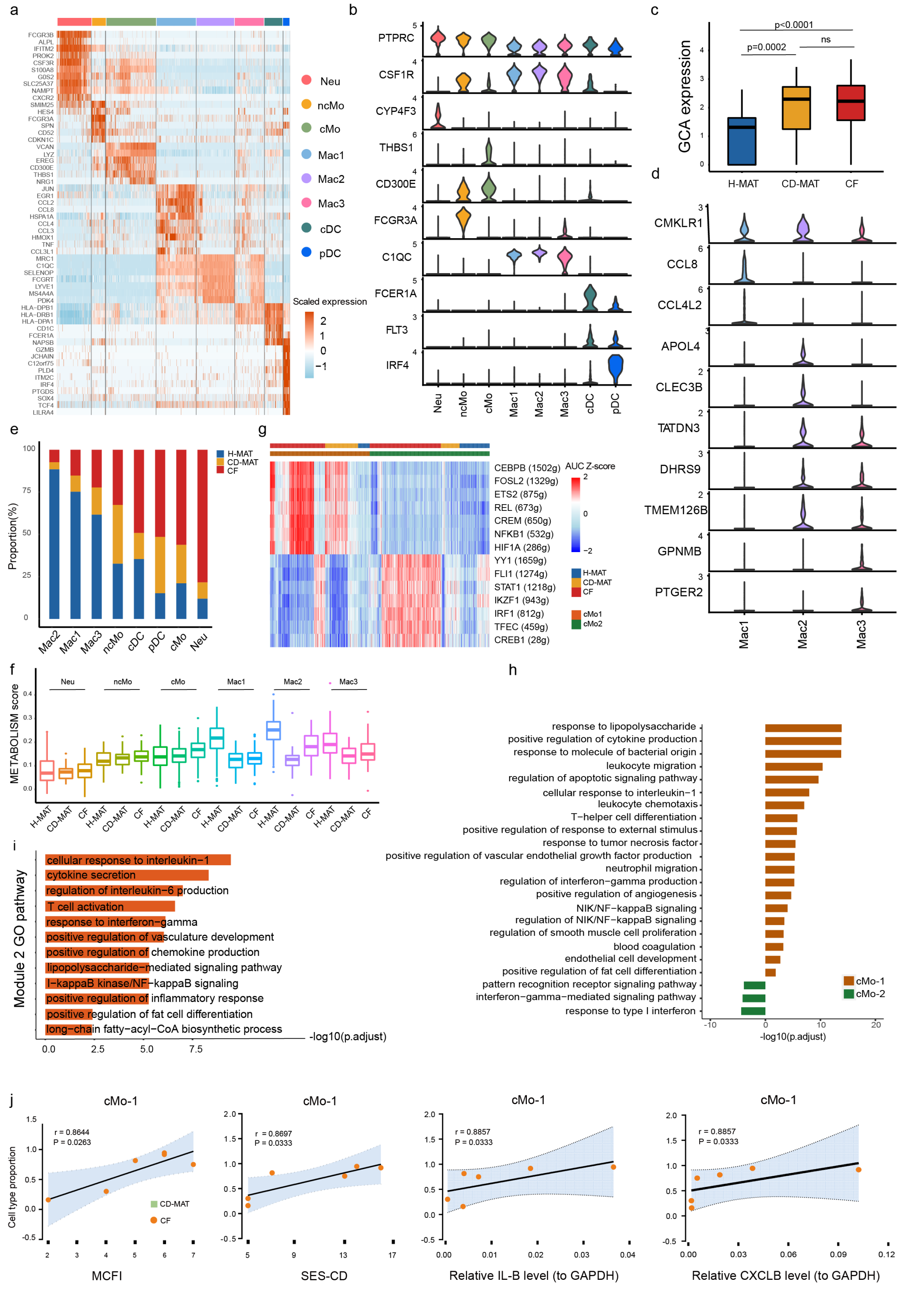

Supplementary Figure 7. Identifying unique myeloid subpopulations in human MAT.
a Heatmap exhibiting the differentially expressed marker genes for each myeloid subpopulation. Cluster identities are shown above the heatmap. Color saturation indicates the strength of expression. **b** Violin plots showing the expression of marker genes for myeloid subpopulations. The y-axis represents log-scaled normalized UMI counts. **c** Boxplots of log-transformed gene expression of GCA in neutrophils from H-MAT (n=2), CD-MAT (n=3) and CF (n=6). **d** Violin plots showing the

expression of marker genes for Mac1-3. The y-axis represents log-scaled normalized UMI counts. **e** Bar plots exhibiting the cellular sources for myeloid subpopulations. Blocks are color-coded by their derived groups. Block heights are proportional to the number of detected cells. **f** The metabolic activity analysis of myeloid subpopulations in H-MAT (n=2), CD-MAT (n=3) and CF (n=6). **g** Heatmap showing expression of representative transcription factors (TFs) in the cMo-1 and cMo-2 subsets across single cells. Columns denote groups and cells, and rows denote genes. **h** GO analysis of the differentially expressed genes between cMo-1 and cMo-2 based on their functions. **i** GO analysis of the averaged expression of all genes in module 2. **j** Estimated proportions of monocytes in scRNA-seq data of CF from 6 individuals plotted against MCFI, SES-CD, as well as expression of IL1B and CXCL8 in strictured intestinal tissues. Linear regression and Spearman's correlation analysis were conducted. Error bands represent a confidence level of 0.95. $P < 0.05$ was considered significant. For boxplot the center line represents the median, box hinges represent first and third quartiles and whiskers represent ± 1.5x interquartile range. **(h,i)** Data were analysed using two-sided Wilcoxon rank sum test. Source data are provided as a Source Data file.

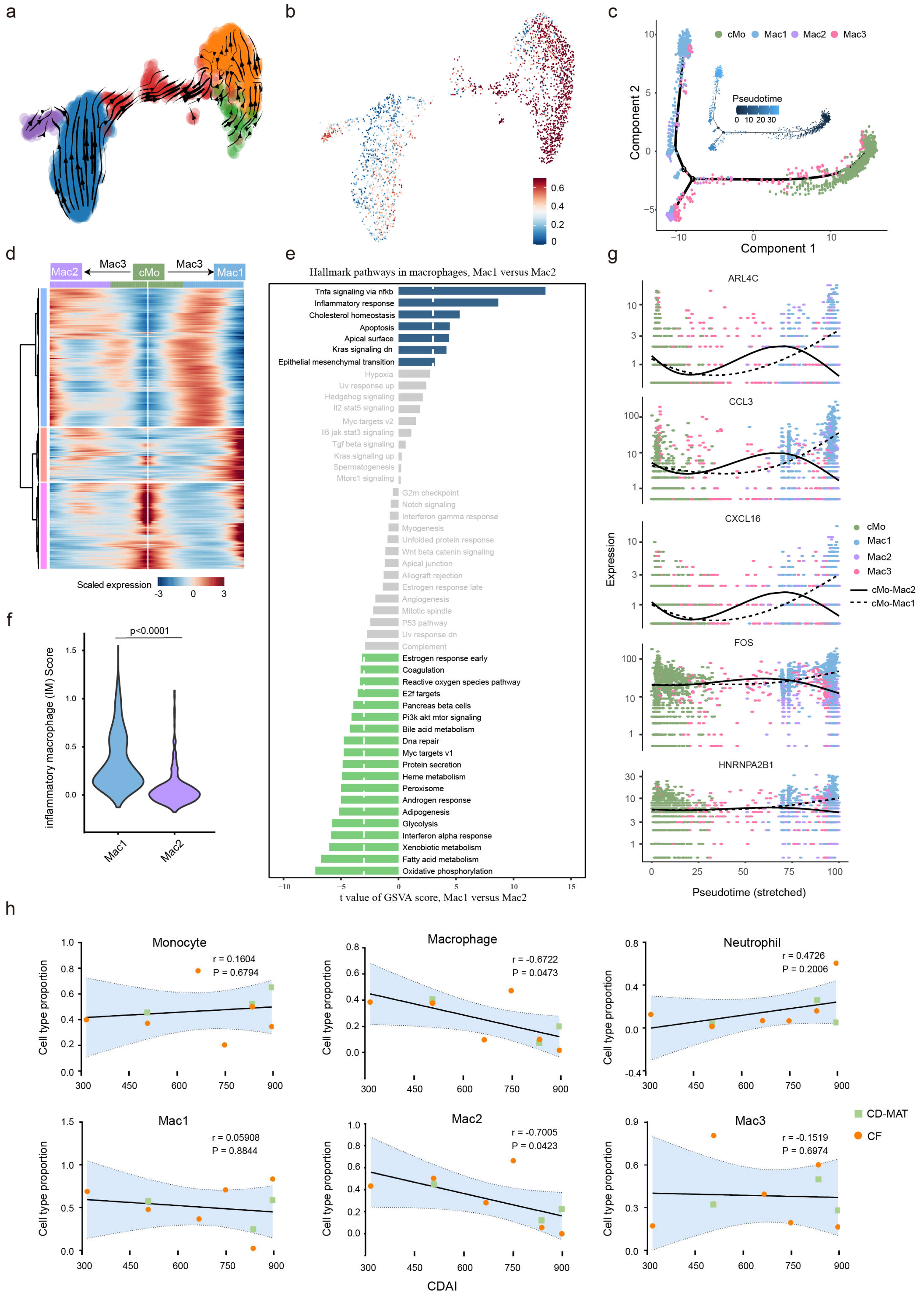

Supplementary Figure 8. Characterization of macrophage subsets.

a RNA-velocity analysis of MAT monocyte and macrophage subsets with the velocity field projected onto the UMAP plot from (Figure 5A). Arrows show the local average velocity evaluated on a regular grid and indicate the extrapolated future states of cells. **b** CytoTRACE scatter plot of human MAT monocyte and macrophage subsets. Colors indicate the level of differentiation from low (blue) to high (red). **c** Monocle analysis of the monocyte and macrophage subsets indicating

pseudotime directionality (internal) and cell type (external); cMo (green), Mac1 (blue), Mac2 (purple) and Mac3 (red). **d** Bifurcation heatmap of enriched genes for Mac2 (left), cMo (middle) and Mac1 (right). Colors indicate increased (red) or decreased (blue) expression. **e** Differences in hallmark pathway activities scored per cell by gene set variation analysis (GSVA), between macrophages isolated from Mac1 or Mac2. T values are from a linear model, corrected for effects from the patient of origin. **f** Inflammatory macrophage gene module score analysis for the Mac subsets based on comparison of signature genes for each cell type from previously defined datasets to differentially expressed genes within each cluster. **g** Gene expression kinetics along pseudotime progression of representative differentially expressed genes between cMo-Mac2 and cMo-Mac1 differentiation trajectories. Solid line: cMo-Mac2 differentiation trajectory, dashed line: cMo-Mac1 differentiation trajectory. **h** Estimated proportions of monocyte, neutrophil and macrophage subsets in scRNA-seq data of CD-MAT or CF from 6 individuals plotted against CDAI or MCFI. Linear regression and Spearman's correlation analysis were conducted. Error bands represent a confidence level of 0.95. $P < 0.05$ was considered significant. Source data are provided as a Source Data file.

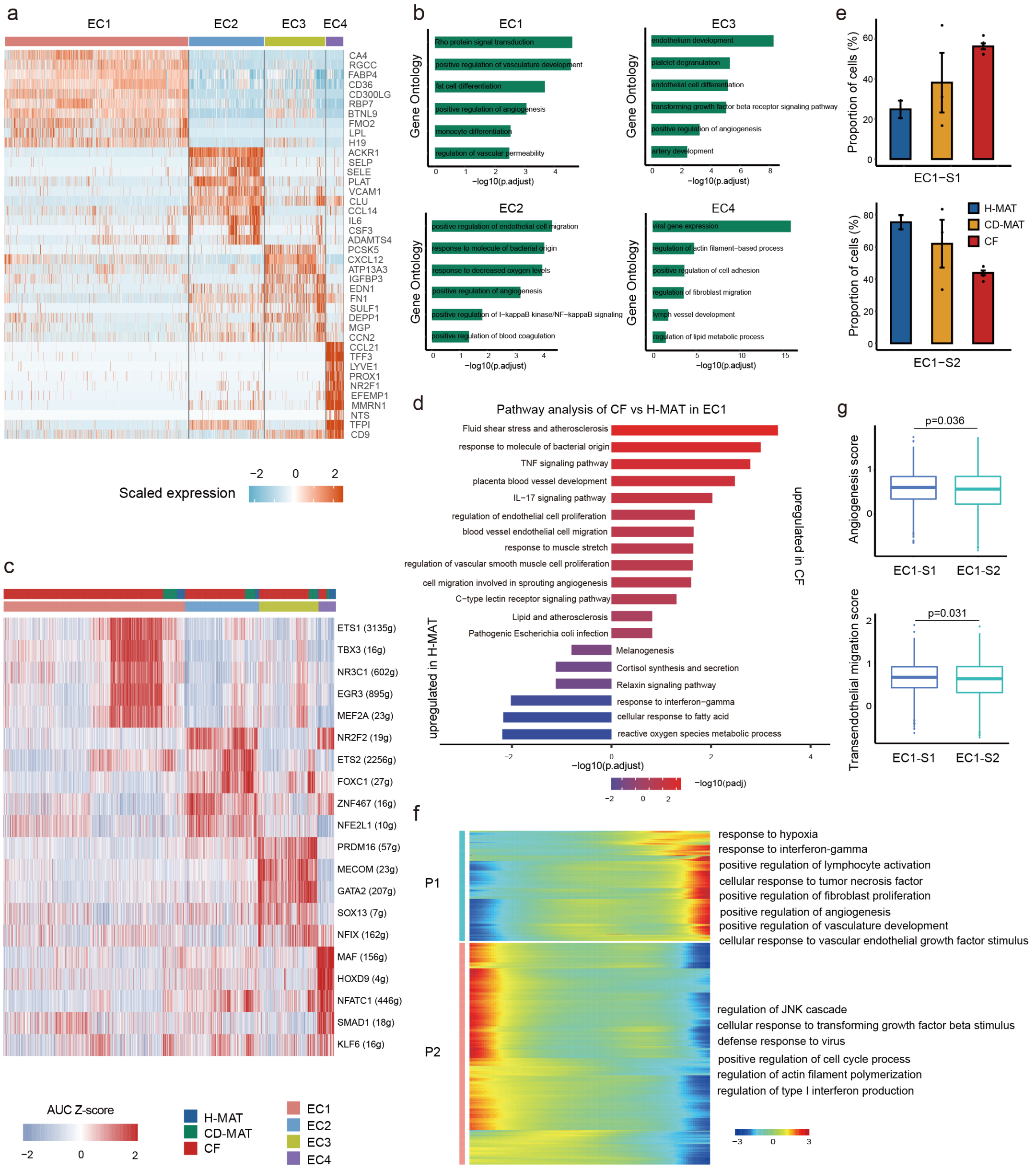

Supplementary Figure 9. Characterization of EC subpopulations in healthy and hyperplastic human MAT.

a Heatmap showing scaled expression of specific marker genes for EC subpopulations. Rows represent genes, and columns represent cells. Colors indicate the expression of genes in each cell. **b** GO analysis of genes significantly enriched in EC subpopulations. **c** Heatmap showing the expression of representative TFs in EC subsets across single cells. Columns denote groups and cells; rows denote genes. **d** Horizontal bar graphs representing differentially expressed pathways in EC1 as assessed by gene set enrichment analysis. **e** Changes in EC1 subsets composition in H-MAT (n=2), CD-MAT (n=3) and CF (n=6) and the data representing mean ±s.d. **f** The differentially expressed genes (rows) along the pseudo-time (columns) is clustered hierarchically into two profiles. The representative gene functions and pathways of each profile are shown. **g** Boxplots comparing the scores of angiogenesis and leukocyte transendothelial migration in selected EC1 subsets in H-MAT (n=2), CD-MAT (n=3) and CF (n=6). For boxplot the center line represents the median, box hinges

represent first and third quartiles and whiskers represent ± 1.5x interquartile range. **(b,d,g)** Data were analysed using two-sided Wilcoxon rank sum test.

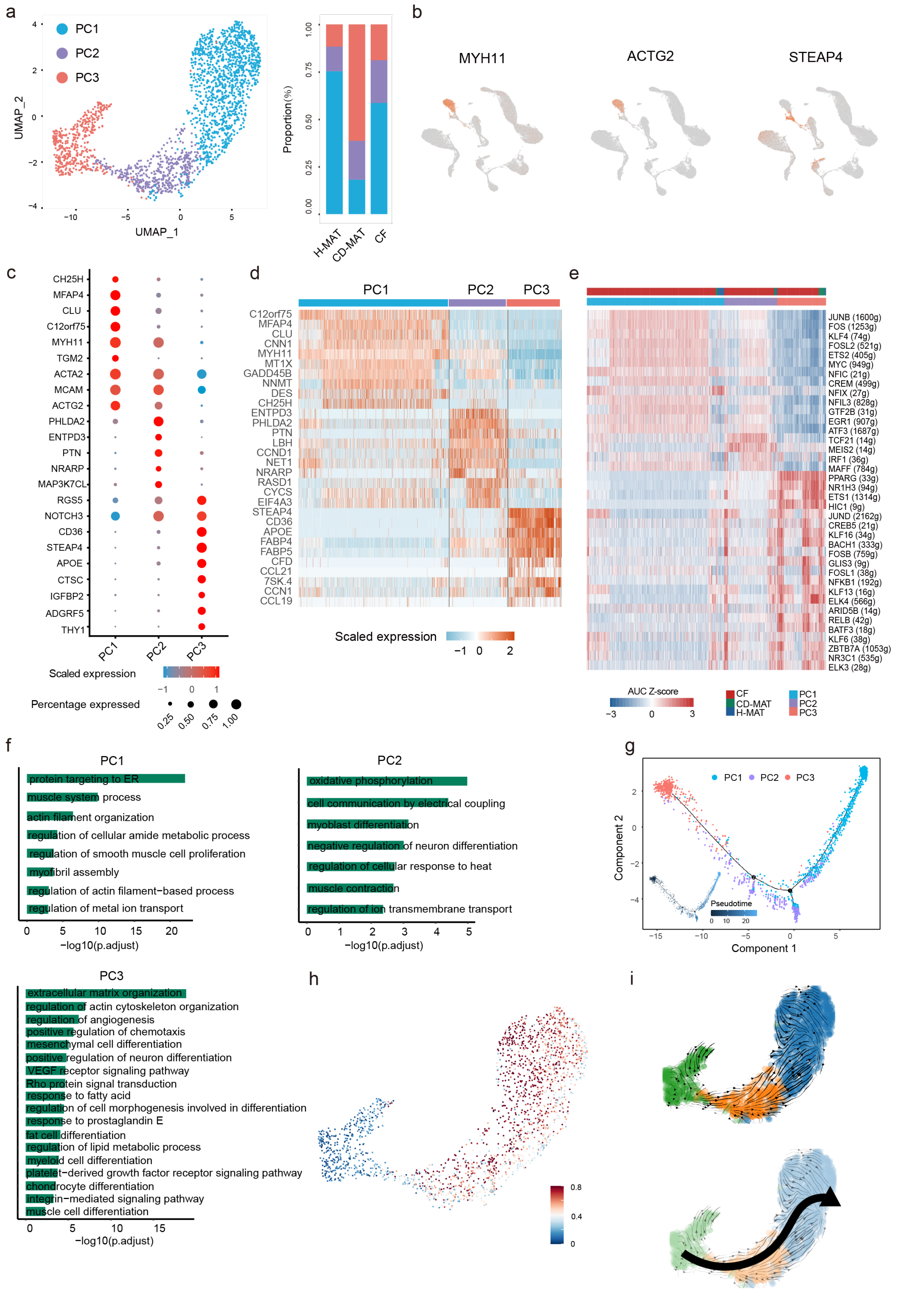

Supplementary Figure 10. Characterization of PC subsets in healthy and hyperplastic human MAT.

a scRNA-seq-generated UMAP plot of pericytes distinguishing three individual subsets. Colored bars indicate the proportion of PCs in H-MAT, CD-MAT and CF. **b** Expression levels of representative pericyte marker genes in each subset are plotted onto the UMAP plot from (Figure. 2A). Color key from gray to red indicates relative expression levels from low to high. **c** Dot plot

showing selected top differentially expressed genes for the PC subsets depicted. Color saturation indicates the strength of expression in positive cells, while dot size reflects the percentage of each cell cluster expressing the gene. **d** Heatmap showing scaled expression of specific marker genes for PC subsets. Rows represent genes, and columns represent cells. Colors indicate the expression of genes in each cell. **e** Heatmap showing the expression of representative TFs in three PC subsets across single cells. Columns denote groups and cells; rows denote genes. **f** GO analysis of genes significantly enriched in PC subsets and p value were generated using two-sided Wilcoxon rank sum test. **g** Monocle analysis of the pericyte populations indicating pseudotime directionality (bottom left) and cell type (middle); PC1 (blue), PC2 (purple) and PC3 (red). **h** CytoTRACE scatter plot of human MAT PC subsets. Color indicates the level of differentiation from low (blue) to high (red). **i** RNA-velocity analysis of MAT pericyte subsets with the velocity field projected onto the UMAP plot of human MAT pericyte subclustered. Small arrows show the local average velocity evaluated on a regular grid and indicate the extrapolated future states of cells (top). Principal curve indicates the manually averaged differentiation directionality projected by RNA-velocity and CytoTRACE analysis (bottom).
