# Supplementary material for: A CCL2+DPP4+ subset of mesenchymal stem cells expedites aberrant formation of creeping fat in humans
Source: Nat Commun. 2023 Sep 20;14:5830. doi: 10.1038/s41467-023-41418-z (PMC10511504; doi:10.1038/s41467-023-41418-z)
Supplement: Supplementary file 3 — Description of Additional Supplementary Files [file 41467_2023_41418_MOESM3_ESM.pdf]

Description of Additional Supplementary Files

Supplementary Data 1
Clinical information for all subjects in our study including 60 patients with Crohn's disease and 27 non-IBD control subjects.

Supplementary Data 2
Lipidomics information.

Supplementary Data 3
Table of differences in lipid metabolites between H-MAT and CF. Descriptive statistics are provided. The variable importance in projection (VIP) was calculated based on the orthogonal partial least squares discriminant analysis (OPLS-DA), and the P-value and fold change (FC) of the nonparametric test were used in combination to screen the differential lipid metabolites. Then, the cut offs of VIP $\geqslant 1$, FC $\geqslant 2$ or FC $\leqslant 0.5$ and $P < 0.05$ were used as standards to screen differential lipid metabolites. The p value were analysed using hypergeometric test.

Supplementary Data 4
Characterization for 8 subjects used in scRNA survey.

Supplementary Data 5
Table of the cluster marker genes for each cell type identified. Descriptive statistics are provided. Data were analysed using two-sided Wilcoxon rank sum test.

Supplementary Data 6
Table of general characteristics of patients used to generate flow cytometry validation experiments. Descriptive statistics are provided for each population.

Supplementary Data 7
Table of the markers' GO enrichment analysis results for each cell type identified. Descriptive statistics are provided. Data were analysed using two-sided Wilcoxon rank sum test.

Supplementary Data 8
Table of the differentially expressed genes between CF and H-MAT. Descriptive statistics are provided. DEG analysis was performed using a two-sided Wilcoxon rank sum test. The Benjamini-Hochberg Procedure was used for adjustments. Data were analysed using two-sided Wilcoxon rank sum test.

Supplementary Data 9
Table of the differentially expressed genes' GO enrichment analysis results for each cell type identified. Descriptive statistics are provided. Data were analysed using two-sided Wilcoxon rank sum test.

Supplementary Data 10
Table of genes in disease and their associated pathways.

Supplementary Data 11
Table of the differentially expressed genes between CD-MAT and H-MAT for each cell type identified. Descriptive statistics are provided. DEG analysis was performed using a two-sided Wilcoxon rank sum test. The Benjamini-Hochberg Procedure was used for adjustments.

Supplementary Data 12
Table of the differentially expressed genes between CF and CD-MAT for each cell type identified. Descriptive statistics are provided. DEG analysis was performed using a two-sided Wilcoxon rank sum test. The Benjamini-Hochberg Procedure was used for adjustments.

Supplementary Data 13
Table of genome-wide association signals for candidate risk genes with CD. Descriptive statistics are provided. Data analysis was performed using a two-sided Wilcoxon rank sum test.

Supplementary Data 14
Table of the cluster marker genes for each MSC subpopulation identified. Descriptive statistics are provided. Data analysis was performed using a two-sided Wilcoxon rank sum test. The Benjamini-Hochberg Procedure was used for adjustments.

Supplementary Data 15
Table of the lineage marker genes and primers.

Supplementary Data 16
Table of the differentially expressed genes for each MSC1 subset identified. Descriptive statistics are provided. DEG analysis was performed using a two-sided Wilcoxon rank sum test. The Benjamini-Hochberg Procedure was used for adjustments.

Supplementary Data 17
List of genes over transition from MSC1 to MSC2 and then MSC3 and the markers' GO enrichment analysis results for the main Module. Data were analysed using two-sided Wilcoxon rank sum test.

Supplementary Data 18
Table of the cluster marker genes for each myeloid cell subpopulation identified. Descriptive statistics are provided. Data analysis was performed using a two-sided Wilcoxon rank sum test. The Benjamini-Hochberg Procedure was used for adjustments.

Supplementary Data 19
Table of the cluster marker genes for each cMo subset identified. Descriptive statistics are provided. Data analysis was performed using a two-sided Wilcoxon rank sum test. The Benjamini-Hochberg Procedure was used for adjustments.

Supplementary Data 20
Table of the interaction of ligand-receptor pairs between cMos and MSCs. Descriptive statistics are provided. Data analysis was performed using a two-sided Wilcoxon rank sum test.

Supplementary Data 21
Table of the cluster marker genes for each EC subpopulation identified. Descriptive statistics are provided. Data analysis was performed using a two-sided Wilcoxon rank sum test. The Benjamini-Hochberg Procedure was used for adjustments.

Supplementary Data 22
Table of the cluster marker genes for each PC subpopulation identified. Descriptive statistics are provided. Data analysis was performed using a two-sided Wilcoxon rank sum test. The Benjamini-Hochberg Procedure was used for adjustments.
