# Supplementary material for: A CCL2+DPP4+ subset of mesenchymal stem cells expedites aberrant formation of creeping fat in humans
Source: Nat Commun. 2023 Sep 20;14:5830. doi: 10.1038/s41467-023-41418-z (PMC10511504; doi:10.1038/s41467-023-41418-z)
Supplement: Supplementary file 5 — Reporting Summary [file 41467_2023_41418_MOESM5_ESM.pdf]

nature portfolio

Corresponding author(s): Lan Bai

Last updated by author(s): 2023-07-31

Reporting Summary

Nature Portfolio wishes to improve the reproducibility of the work that we publish. This form provides structure for consistency and transparency in reporting. For further information on Nature Portfolio policies, see our Editorial Policies and the Editorial Policy Checklist.

Statistics

For all statistical analyses, confirm that the following items are present in the figure legend, table legend, main text, or Methods section.

n/a	Confirmed	
☐	☒	The exact sample size (*n*) for each experimental group/condition, given as a discrete number and unit of measurement
☐	☒	A statement on whether measurements were taken from distinct samples or whether the same sample was measured repeatedly
☐	☒	The statistical test(s) used AND whether they are one- or two-sided<br>*Only common tests should be described solely by name; describe more complex techniques in the Methods section.*
☐	☒	A description of all covariates tested
☐	☒	A description of any assumptions or corrections, such as tests of normality and adjustment for multiple comparisons
☐	☒	A full description of the statistical parameters including central tendency (e.g. means) or other basic estimates (e.g. regression coefficient) AND variation (e.g. standard deviation) or associated estimates of uncertainty (e.g. confidence intervals)
☐	☒	For null hypothesis testing, the test statistic (e.g. *F*, *t*, *r*) with confidence intervals, effect sizes, degrees of freedom and *P* value noted<br>*Give P values as exact values whenever suitable.*
☒	☐	For Bayesian analysis, information on the choice of priors and Markov chain Monte Carlo settings
☒	☐	For hierarchical and complex designs, identification of the appropriate level for tests and full reporting of outcomes
☒	☐	Estimates of effect sizes (e.g. Cohen's *d*, Pearson's *r*), indicating how they were calculated

Our web collection on statistics for biologists contains articles on many of the points above.

Software and code

Policy information about availability of computer code

Data collection	Lipid contents were detected by LC–MS/MS (AB Sciex QTRAP 6500, Framingham, USA). Flow cytometric experiments were carried out with a BD FACSAria (BD Biosystems). Images were captured using either ZEISS LSM880 or Olympus BX-53 microscope (Olympus). The scRNA-seq libraries were generated using the Chromium Single Cell 3′ v.3 assay (10× Genomics). R programming language (version 4.1.1) was applied for statistical analyses and the generation of graphs.
Data analysis	Monocle2 R package (v2.14.0) was used to perform trajectory analysis. Flow cytometry data were analysed by Flow Jo (Ashland, OR, V10.8.1). CytoTRACE analysis was carried out using the CytoTRACE R package (v0.3.3) with the raw counts for cells in the intended cell types and default parameters. The resulting CytoTRACE score for each cell was plotted on the UMAP. RNA velocity analysis was performed using dynamical modelling with the package scVelo(v0.2.3) to analyze cellular trajectory. Statistical significance was assessed using Prism 8.0 software (GraphPad Software). Pathway analyses were exported using the GSEABase package (v1.48.0). For cell proliferation assay, the number of cells was counted with a SpectraMax M4 (Molecular Devices) under absorbance at A450. For scRNA-seq, the cellranger count (v5.0.1) was used to name the cells, while the SoupX R package (v1.5.2), the DoubletFinder R package (v2.0.3) and the Seurat R package (v4.0.2) were used to the quality control of scRNA-seq data. ClusterProfiler (v3.14.3) R package was used for GO enrichment analysis. GRNBoost2 (https://github.com/tmoerman/arboreto) in pySCENIC (v0.10.1) was applied to infer gene regulatory networks from raw count data. CellPhoneDB (v2.1.7) was applied for ligand–receptor analysis, and the number of interactions between each pair of cell types was plotted by Cytoscape (v3.7.0). GSVA package (v1.34.0) and the limma package (v3.46.0) were used for Gene set variation analysis. The scMetabolism package (v0.2.1) was used to compute the Metabolic pathway scores for each subcluster of cells were averaged and visualized using the ggplot2 R package.single-cell metabolic activity of MSCs and myeloid subtype cells.

For manuscripts utilizing custom algorithms or software that are central to the research but not yet described in published literature, software must be made available to editors and reviewers. We strongly encourage code deposition in a community repository (e.g. GitHub). See the Nature Portfolio guidelines for submitting code & software for further information.

Data

Policy information about availability of data

All manuscripts must include a data availability statement. This statement should provide the following information, where applicable:

- Accession codes, unique identifiers, or web links for publicly available datasets
- A description of any restrictions on data availability
- For clinical datasets or third party data, please ensure that the statement adheres to our policy

The datasets (raw data) generated in this study are available through the Sequence Read Archive (SRA), BioProject ID: PRJNA871957. The processed single cell count tables are provided in Gene Expression Omnibus; GSE215001 (https://www.ncbi.nlm.nih.gov/geo/query/acc.cgi?acc=GSE215001). The Molecular Signatures Database (MSigDB) can be explored at https://www.gsea-msigdb.org/gsea/msigdb. Source data are provided with this paper.

Research involving human participants, their data, or biological material

Policy information about studies with human participants or human data. See also policy information about sex, gender (identity/presentation), and sexual orientation and race, ethnicity and racism.

Reporting on sex and gender	The findings from our study apply to both males and females.<br><br>Sex was considered in study design. The detail is as shown in (Methods: Patients and specimen preparation and Supplementary Table 1a).<br><br>Sex was self-reported, which is the biological sex, as validated in personal ID that is regarded as an extremely accurate record for sex in the country (China) where the samples were collected. The medical management system of the Nanfang Hospital of Southern Medical University was used to obtain this information.<br><br>The source data disaggregated by sex, actually by each individual, was provided. The consent for sharing individual-level data has been obtained. A total of 87 samples were collected in this study, with the ratio of male to female as of 56: 31.<br><br>We performed a sex-based analysis on the targeted data, and no difference was found.
Reporting on race, ethnicity, or other socially relevant groupings	All the individuals in this study are of Asian descent, specifically Han Chinese.
Population characteristics	The patients were diagnosed as CD before resection; this diagnosis was confirmed by pathological examination with the specimens from the patients after resection.<br><br>The patients enrolled in the control group underwent resection for non-inflammatory bowel disease ileal surgery. All participants in this study were of Han ethnicity.<br><br>For the scRNA-seq analysis, 8 patients were enrolled, including 5 males with a mean age of 33.8 years and 3 females with a mean age of 59 years.<br><br>Additionally, for the lipidomics analysis, 37 patients were enrolled, including 21 males and 16 females, with a mean age of 41.5 and 47.3 years, respectively.<br><br>The detail is as shown in Supplementary Table 1a.
Recruitment	The donors were recruited by Nanfang Hospital or the Sixth Affiliated Hospital of Sun Yat-sen University. Subjects were recruited chronologically, with no specific inclusion and exclusion criteria. A total of 60 patients who were diagnosed as CD and 28 patients who underwent resection in non-inflammatory bowel disease ileal surgery were enrolled in this study.
Ethics oversight	The studies were approved by the local ethical committee (NFEC-2021-053) and informed consent was obtained from the participant.

Note that full information on the approval of the study protocol must also be provided in the manuscript.

Field-specific reporting

Please select the one below that is the best fit for your research. If you are not sure, read the appropriate sections before making your selection.

☒ Life sciences ☐ Behavioural & social sciences ☐ Ecological, evolutionary & environmental sciences

For a reference copy of the document with all sections, see nature.com/documents/nr-reporting-summary-flat.pdf

Life sciences study design

All studies must disclose on these points even when the disclosure is negative.

Sample size	Sample size was determined based on the complexity, cost of the experiments and sample availability.<br>For FACS data: Mesenchymal stem (MSC) cells (fresh samples): CF (n=8), paired CD-MAT (n=8) and H-MAT (n=10). Pericytes (fresh samples): CF (n=6), paired CD-MAT (n=6) and H-MAT (n=3). Endothelial cells (fresh samples): CF (n=5), paired CD-MAT (n=5) and H-MAT (n=3). MSC subpopulations (fresh samples): CF (n=6), paired CD-MAT (n=6) and H-MAT (n=4).<br>MSC cells (fresh samples): CF (n=6), paired CD-MAT (n=6) and H-MAT (n=4).<br>For analysis of lipid metabolites: ≥20mg adipose tissue (frozen samples) CF (n=20), paired CD-MAT (n=18) and H-MAT (n=15).<br>For scRNA-seq: Stromal vascular fraction (SVF) cells (fresh sample): CF (n=6), paired CD-MAT (n=3) and H-MAT (n=2).
Data exclusions	No data was excluded.
Replication	The number of experiments performed for each panel is indicated in Figure legend. Data were replicated with independent cohort of patients.
Randomization	Randomization was not performed for this study.
Blinding	Blinding was not required for this study because it was critical to identify if samples were control or CD patient to interpret the data.

Reporting for specific materials, systems and methods

We require information from authors about some types of materials, experimental systems and methods used in many studies. Here, indicate whether each material, system or method listed is relevant to your study. If you are not sure if a list item applies to your research, read the appropriate section before selecting a response.

Materials & experimental systems

n/a	Involved in the study	
☐	☒	Antibodies
☒	☐	Eukaryotic cell lines
☒	☐	Palaeontology and archaeology
☒	☐	Animals and other organisms
☒	☐	Clinical data
☒	☐	Dual use research of concern
☒	☐	Plants

Methods

n/a	Involved in the study	
☒	☐	ChIP-seq
☐	☒	Flow cytometry
☒	☐	MRI-based neuroimaging

Antibodies

Antibodies used	Immunohistochemistry and immunofluorescence: CD31 (1 µg/ml; Mouse monoclonal; Abcam, cat: ab9498), CD26 (diluted 1:200; Rabbit monoclonal; DPP4, Abcam, cat: ab215711), CD142 (diluted 1:100; Rabbit monoclonal; F3, Abcam, cat: ab228968), CD54 (1000 µg/ml; Mouse monoclonal; ICAM1, Proteintech, cat: 60299), CD146 (diluted 1:200; Rabbit monoclonal; Abcam, cat: ab757 69), CA4 (diluted 1:200; Rabbit Polyclonal; Proteintech, cat: 13931), Hoechst 33342 (12.3 mg/ml; Thermo Fisher cat: 62249), Goat Anti-Rabbit IgG （HRP） (diluted 1:4000, Abcam, cat: ab205718), Alexa Fluor®594 donkey anti-mouse lgG (H+L) (diluted 1:400, life technologies, cat: A21203) and Alexa Fluor®647 donkey anti-Rabbit lgG (H+L) (diluted 1:200, Abcam, cat: ab150075).<br>Flow Cytometry: Anti-human CD31 (diluted 1:100; PE; BD Biosciences, cat:303106), CD45 (diluted 1:100; FITC; BD Biosciences, cat:555482), CD26 (diluted 1:100; R718; BD Biosciences, cat:752183), CD54 (diluted 1:100; BV605; BD Biosciences, cat:740404), CD142 (diluted 1:100; BV421; BD Biosciences, cat:744003), CD146 (diluted 1:100; BV510; BioLegend, cat:361021), MCP-1 (diluted 1:100; APC; BioLegend, cat:502612) and Fixable Viability Stain 780 (diluted 1:500; APC/cy7; BD Biosciences, cat: 565388).<br>Magnetic-activated cell sorting: FcR Blocking Reagent (Miltenyi Biotec, cat: 130-091-935), CD31-Microbeads (Miltenyi Biotec, cat: 130-091-935), CD45-Microbeads (Miltenyi Biotec, cat: 130-045-801), anti-142 (Miltenyi Biotec, cat: 130-115-682), anti-CD54 (Biolegend, cat: 322706), anti-CD26 (Biolegend, cat: 302718), anti-biotin microbeads (Miltenyi Biotec, cat: 130-090-485), anti-CD98 (Miltenyi Biotec, cat: 130105550). A concentration of 1 × 108 cells per millilitre were incubated with 5 µg of each biotin-conjugated antibody.
Validation	All antibodies were bought from commercial suppliers and are validated by the vendor for the species and assay used in our study. Validated data is available on the vendors' websites.

Flow Cytometry

Plots

Confirm that:

☒ The axis labels state the marker and fluorochrome used (e.g. CD4-FITC).

☒ The axis scales are clearly visible. Include numbers along axes only for bottom left plot of group (a 'group' is an analysis of identical markers).

☒ All plots are contour plots with outliers or pseudocolor plots.

☒ A numerical value for number of cells or percentage (with statistics) is provided.

Methodology

Sample preparation	For testing nuclear markers in MSC1-S1s, cells were resuspended in 1 mL fixation/permeabilization working solution (Invitrogen; cat: 00-5523-00). After 30 min in the dark, 2 mL of 1X permeabilization buffer was added and then subjected to centrifugation at 1000 rpm at RT for 5 min. Subsequently, the cells were stained with MCP-1 (APC; BioLegend, cat: 502612) for 30 min in the dark and washed with 2 mL of 1X permeabilization buffer. Then, doublets and dead cells were excluded based on forwards and side scatter.
Instrument	All flow cytometric experiments were performed by BD FACSAria (BD Biosystems).
Software	Flow cytometry data were analysed by Flow Jo (Ashland, OR).
Cell population abundance	Cell sorting purity was between 98-99% in all experiments.
Gating strategy	The following gating strategy was applied to analyse the cells: doublets and dead cells were excluded based on forwards and side scatter. Immune cells were purified using CD45-FITC, and ECs were identified using CD31-PE. Gates were created to collect MSCs (CD31−CD45−CD146−) and PCs (CD31−CD45−CD146+). Then, MSC1s (CD26+CD142−CD54−), MSC2s (CD26−CD142+CD54−) and MSC3s (CD26−CD142−CD54+) were identified from MSCs. MSC1-S1s were identified from MSC1s by staining with MCP-1-APC.

☒ Tick this box to confirm that a figure exemplifying the gating strategy is provided in the Supplementary Information.
